# The impact of inappropriate antibiotics on bacteremia patients in a community hospital in Taiwan: an emphasis on the impact of referral information for cases from a hospital affiliated nursing home

**DOI:** 10.1186/1471-2334-13-500

**Published:** 2013-10-24

**Authors:** Chih-Jen Yang, Yu-Chieh Chung, Tun-Chieh Chen, Hsu-Liang Chang, Ying-Ming Tsai, Ming-Shyan Huang, Yen-Hsu Chen, Po-Liang Lu

**Affiliations:** 1 Department of Internal Medicine, Kaohsiung Municipal Ta-Tung Hospital, Kaohsiung Medical University Hospital, Kaohsiung Medical University, Kaohsiung, Taiwan; 2 Division of Pulmonary and Critical Care Medicine, Kaohsiung Medical University, Kaohsiung, Taiwan; 3 Division of Infectious Diseases, Department of Internal Medicine, Kaohsiung Medical University Hospital, Kaohsiung, Taiwan; 4 Wen-Hsiung Hospital, Kaohsiung, Taiwan; 5 College of Medicine, Kaohsiung Medical University, Kaohsiung, Taiwan; 6 Department of Internal Medicine, Kaohsiung Medical University Hospital, Kaohsiung Medical University, #100, Tzyou 1st Road, Kaohsiung City 807, Taiwan

**Keywords:** Bacteremia, Nursing home, Inappropriate antibiotics, Community hospital

## Abstract

**Background:**

Evidence for the impact of inappropriate antimicrobial therapy on bacteremia is mainly from studies in medical centers. We investigated the impact of inappropriate antimicrobial therapy on bacteremia in a community hospital. In particular, patients from the hospital’s affiliated nursing home were sent to the hospital with adequate referral information.

**Methods:**

We performed a retrospective study to collect data of patients with bacteremia in a community hospital in Taiwan from 2005 to 2007.

**Results:**

A total of 222 patients with blood stream infection were diagnosed, of whom 104 patients (46.8%) died. The rate of initial inappropriate antibiotic prescriptions was high (59%). Multivariate analysis revealed that patients with initial inappropriate antibiotics, patients with ventilator support and patients requiring ICU care were the independent predictors for inhospital mortality. Patients referred from the hospital-affiliated nursing home and patients with normal WBC counts had better survival outcome. More than 80% cases infected with methicillin-resistant *Staphylococcus aureus* (MRSA) and *Enterococcus faecalis* received initial inappropriate antimicrobial therapy. With the longer delay to administer appropriate antibiotic, a trend of higher mortality rates was observed.

**Conclusions:**

Bacteremia patients from a hospital-affiliated nursing home had a better prognosis, which may have been due to the adequate referral information. Clinicians should be aware of the commonly ignored drug resistant pathogens, and efforts should be made to avoid delaying the administration of appropriate antibiotic therapy.

## Background

Blood stream infections (BSI) are a major cause of morbidity and mortality, and they are also associated with an increased one-year mortality rate [1]. Two important factors for the decreased efficacy of antimicrobial therapy are treatment with inappropriate antimicrobial agents and the delay of appropriate antimicrobial therapy [2]. However, the increasing antimicrobial resistance of micro-organisms has decreased the likelihood of patients receiving appropriate empiric antimicrobial therapy.

Many studies have reported that inappropriate empirical antimicrobial treatment for BSI resulted in higher patient morbidity and mortality rates and increased healthcare costs [1,3-11]. Most of these studies were conducted in medical centers and university hospitals. However, other studies have failed to find a relationship between mortality and initial inappropriate antibiotic treatment in BSI patients [12,13]. The conflicting findings may be due to different rates of inappropriate antimicrobial therapy, the case number in each study, differences in disease severity and comorbidities of the patients, and differences in hospital care systems [14]. It is therefore interesting to investigate the influencing factors in different hospital settings and healthcare systems.

Studies of the impact of inadequacy of antibiotics on BSI patients in medical centers in Taiwan have been reported [3,6,15]. However, no studies have focused on community hospital setting. In addition, the correlation of BSI outcomes and different BSI case sources (communities, nursing homes, and hospitals) has never been documented. A current trend in Taiwan is for a hospital to have an affiliated or contracted nursing home that may or may not include respiratory care wards. It is possible for BSI patients who are referred from an affiliated nursing home to the parent hospital to have an advantage over patients admitted directly to the hospital for BSI because healthcare workers of the affiliated nursing home provide patients’ medical information. However, this has not been thoroughly studied before.

We performed this retrospective study in a community hospital in Taiwan. The hospital received cases from the community, including those from the hospital affiliated nursing home with which there was good communication and 24-hour physician coverage. All of the patients residing at the nursing home were sent to the parent hospital when they became ill.

The aim of this study was to analyze the impact of initial inappropriate or appropriate antibiotic treatment, focusing on mortality, pathogens and clinical parameters. We also investigated the epidemiology, microbiology and prognosis in community acquired, nursing home acquired and hospital acquired blood stream infections.

## Methods

### Study location and patient selection

This study was conducted at Wen-Hsiung Hospital, which is a community hospital located in Kaohsiung, Taiwan. There is a 20-bed respiratory care ward, a 6-bed intensive care unit and a 26-bed ordinary ward in the hospital. Patients in a 128-bed nursing home which is affiliated to Wen-Hsiung Hospital are sent to the hospital when medical needs arise. Patients in the affiliated nursing home are cared for by at least one staff physician from the community hospital either in-house or on-call at all times. For there was no Institute Ethics Committee at the community hospital, this work was approved by the Institute Ethics Committee of Kaohsiung Medical University Hospital (KMUH-IRB-2013003) according to Taiwan national regulation.

All cases of bacteremia were divided into three sources: community acquired, hospital acquired and nursing home acquired. The patients were identified by review of microbiology laboratory records from January 1, 2005 through December 31, 2007. They were included in the study if their blood cultures were drawn either at the hospital or in the emergency department immediately prior to admission, and if the culture results were positive for a fungus or bacteria. Patients younger than 18 years and patients who visited the emergency department but were not hospitalized were excluded. All case records were reviewed by two physicians (Dr. Yang CJ and Dr. Chung YC). All of the clinical and mortality data were obtained from the patients’ medical records as well as their charts.

### Definition and data collection

Bloodstream infections was diagnosed by the presence of clinical or laboratory evidence of sepsis. The definition of sepsis was based on the ACCP/SCCM consensus conference committee [16]. The possibility of a contaminated blood culture was determined after agreement by two doctors. A community-acquired BSI was defined when bacteremia occurred within the first 48 hours of hospital admission for patients from the local community. A nursing home-acquired BSI was defined when it occurred at the time of hospital admission or within 48 hours of admission for patients who came from the hospital affiliated nursing home. A hospital-acquired BSI was defined when it occurred more than 48 hours after the beginning of a period of hospitalization. Patients who were transferred from other hospitals or other nursing homes were excluded from the study. In addition, polymicrobial blood stream infections were also excluded.

Mortality was the main outcome. Appropriate antibiotic therapy was defined as the initial antimicrobial agent being active in vitro against the infecting organism, and if the drug was given at an appropriate dose and by appropriate route of administration. Initial therapy was defined as the antibiotics received on the first day of therapy for the BSI. Time to appropriate antibiotic administration was recorded in the inappropriate therapy group.

Information with regards to comorbid medical conditions such as diabetes mellitus, malignancy, chronic obstructive pulmonary disease, liver cirrhosis, ventilator support, end stage renal disease, vascular disease (including coronary artery disease and cerebral vascular disease) were documented. The other recorded variables were age, sex, source of infection, leukocytes, use of catheters, tracheostomy status, steroid use, postoperative status, length of stay more than 30 days, hospitalized site, sepsis status (sepsis, severe sepsis, septic shock and multiple organ dysfunction syndrome), microorganism isolates, CURB-65 score items including confusion, blood urine nitrogen > 20 mg/dl, shock status (systolic blood pressure > 90 mmHg or diastolic pressure < 60 mmHg), respiratory rate > 30 per minute) [17] and the different sources include community, hospital and the hospital affiliated nursing home. The referral medical information from nursing homes included hospitalization history, previous culture report, and recent infection sites, Antimicrobial susceptibility testing of the BSI isolates was performed according to the National Committee for Clinical Laboratory standards [18].

### Statistical analysis

Descriptive statistics were implemented to summarize the patient characteristics as mean (SD) or proportions. The chi-square or Fisher’s exact test for the categorical data and the Student’s *t*-test or Mann–Whitney *U* test for the continuous data were used to compare data between different groups. In the multivariaate analysis, we analyzed variables with a *P* value <0.05 from the univariate analysis. To control for potential confounding factors, a multinominal logistic regression analysis was performed evaluating the possible covariates. A *P* value of less than 0.05 was considered to be statistically significant. SPSS version 14.0 software (SPSS Inc., Chicago, IL, USA) was used for data analysis.

## Results

Between January 1, 2005 and December 31, 2007, a total of 222 cases of BSI were diagnosed at the hospital. Thirty-six patients had community-acquired, 129 patients had hospital-acquired, and 57 patients had nursing home-acquired BSI. The initial inappropriate antibiotic prescription rate was 59% (131/222). One hundred and four patients (46.8%) died during their hospitalization. Among the three sources, hospital-acquired acquired bacteremia had the worst prognosis with a mortality rate of 58.91%, and patients with nursing home-acquired BSI had the best prognosis, with a mortality rate of 26.3%. In the nursing home-acquired group, the four most common pathogens were *Escherichia coli* (16 cases, 28.07%), *Klebsiella pneumoniae* (5 cases, 8.77%), *Proteus mirabilis* (5 cases, 8.77%) and Methicillin susceptible *Staphylococcus aureus* (MSSA) (5 cases, 8.77%). The most common sources of infection were urinary tract infections (23 cases, 40.35%) and respiratory tract infections (20 cases, 35.08%).

Univariate analysis of the demographics, comorbidities, laboratory data, sources of infection, sepsis status, admission site and adequacy of the antibiotic treatment with regards to survival and mortality is present in Table 1 and Table 2. Hospital mortality was correlated with older age, higher serum creatinine, lower serum albumin, more ventilator support and more central venous catheterization. The mortality rate was higher in those with a serum white blood cell count > 20 × 10^3^/ul, length of hospital stay > 30 days, hospitalized in the intensive care unit, with septic shock, confused status, blood urine nitrogen > 20 mg/dL, systolic BP < 90 mmHg or diastolic BP < 60 mmHg, nosocomial infection and inappropriate antibiotic treatment. In contrast, those with a normal white blood cell count (between 4 × 10^3^ to 10 × 10^3^/ul), admitted to the ordinary ward, sepsis status and with nursing home-acquired BSI were more likely to survive.

**Table 1:** The demographics, co-morbidities and medical devices used when the bacteremia occurred of the inhospital survivors and non-survivors

**Factors**	**Survivors (n = 118)**	**Non-survivors (n = 104)**	**OR**	**95% CI**	***P *** **value**
Age	68.78+/-16.65	74.88+/-11.07			<0.01
Male gender	74 (62.7%)	55 (52.9%)	0.667	0.390-1.141	0.18
DM	53 (44.9%)	54 (51.9%)	1.325	0.781-2.247	0.36
Malignancy	15 (12.7%)	15 (14.4%)	1.157	0.536-2.499	0.86
LC	9 (7.6%)	7 (6.7%)	0.874	0.314-2.436	1.00
Ventilator use	41 (34.7%)	65 (62.5%)	3.130	1.808-5.419	<0.01
ESRD	14 (11.9%)	27 (26.0%)	2.605	1.281-5.296	0.01
COPD	31 (26.3%)	19 (18.3%)	0.627	0.329-1.195	0.21
Vascular Disease	79 (66.9%)	72 (69.2%)	1.111	0.631-1.957	0.83
Transplantation	0	0			
Foley	53 (44.9%)	57 (54.8%)	1.487	0.876-2.526	0.18
Tracheostomy	50 (42.4%)	58 (55.8%)	1.715	1.007-2.919	0.06
CVC	37 (31.4%)	56 (53.8%)	2.554	1.477-4.416	<0.01
Hemocatheter	6 (5.1%)	11 (10.6%)	2.208	0.787-6.197	0.20
Steroid	5 (4.2%)	5 (4.8%)	1.141	0.321-4.059	1.00
Post-OP	1 (0.8%)	1 (1.0%)	1.136	0.070-18.391	1.00

**Table 2:** The laboratory data, sources of infection, sepsis status, admission site and initial inappropriate antibiotic use among the inhospital survivors and non-survivors

**Factors**	**Survivors (n = 118)**	**Non-survivors (n = 104)**	**OR**	**95% CI**	***P *** **value**
WBC ( /uL)					
<4000	8 (7.0%)	6 (5.9%)	0.828	0.277-2.473	0.95
4000-10000	42 (36.8%)	16 (15.7%)	0.319	0.166-0.614	<0.01
10000-20000	50 (43.9%)	51 (50.0%)	1.280	0.749-2.188	0.44
>20000	14 (12.3%)	29 (28.4%)	2.838	1.401-5.746	<0.01
Creatinine	1.68 ± 1.35	2.56 ± 1.56			<0.01
Albumin	3.08 ± 0.53	2.84 ± 0.52			<0.01
C-reactive protein	10.10 ± 7.15	13.39 ± 7.92			0.03
Thrombocytopenia	6 (5.1%)	5 (4.8%)	0.943	0.279-3.184	1.00
Pulmonary infection	25 (21.2%)	33 (31.7%)	1.729	0.945-3.165	0.10
UTI	39 (33.1%)	26 (25.0%)	0.675	0.376-1.214	0.24
IAI	8 (6.8%)	1 (1.0%)	0.133	0.016-1.086	0.04
SSTI	8 (6.8%)	9 (8.7%)	1.303	0.483-3.510	0.79
SWI	0	0			
CRBSI	14 (11.9%)	14 (13.5%)	1.156	0.523-2.553	0.88
CNS infection	0	0			
Unknown infection focus	24 (20.3%)	23 (22.1%)	1.112	0.584-2.119	0.87
Initial inappropriate antibiotic use	59 (50.0%)	72 (69.2%)	2.250	1.297-3.904	<0.01
Sepsis	84 (71.2%)	34 (32.7%)	0.197	0.111-0.348	<0.01
Severe Sepsis	20 (16.9%)	20 (19.2%)	1.167	0.588-2.314	0.79
Septic Shock	9 (7.6%)	44 (42.3%)	8.881	4.06-19.44	<0.01
MODS	-	5 (4.8%)	-	-	0.02
Confusion	79 (66.9%)	96 (92.3%)	5.924	2.62-13.41	<0.01
RR >30 (per minute)	27 (22.9%)	34 (32.7%)	1.637	0.904-2.964	0.14
BUN >20 (mg/dl)	79 (66.9%)	92 (88.5%)	3.785	1.854-7.725	<0.01
SBP <90 mmHg or DBP <60 mmHg	17 (14.4%)	52 (50.0%)	5.941	3.13-11.29	<0.01
Community - acquired	24 (20.3%)	12 (11.5%)	0.511	0.241-1.082	0.11
Nursing home – acquired	42 (35.6%)	15 (14.4%)	0.305	0.157-0.593	<0.01
Hospital – acquired	53 (44.9%)	76 (73.1%)	3.329	1.892-5.857	<0.01
ICU stay	17 (14.4%)	45 (43.3%)	4.531	2.380-8.626	<0.01
LOS > 30 days	34 (28.8%)	51 (49.0%)	2.377	1.367-4.136	<0.01

*WBC* White cell counts, *UTI* Urinary tract infection, *IAI* Intra-abdominal infection, *SSTI* Superficial soft infection, *CRBSI* Catheter related blood stream infection, *CNS* Central venous infection, *CVC* Central venous catheter, *OP* Operation, *LOS* Length of stay, *MODS* Multiple organ dysfunction syndrome, *RR* Respiratory rate, *BUN* Blood urea unit, *SBP* Systolic blood pressure, *DBP* Diastolic blood pressure. Except for two variables including LOS > 30 days and *ICU* stay (ICU care is required during hospitalization), all the variables were measured when bacteremia occurred.

**Figure 1:**
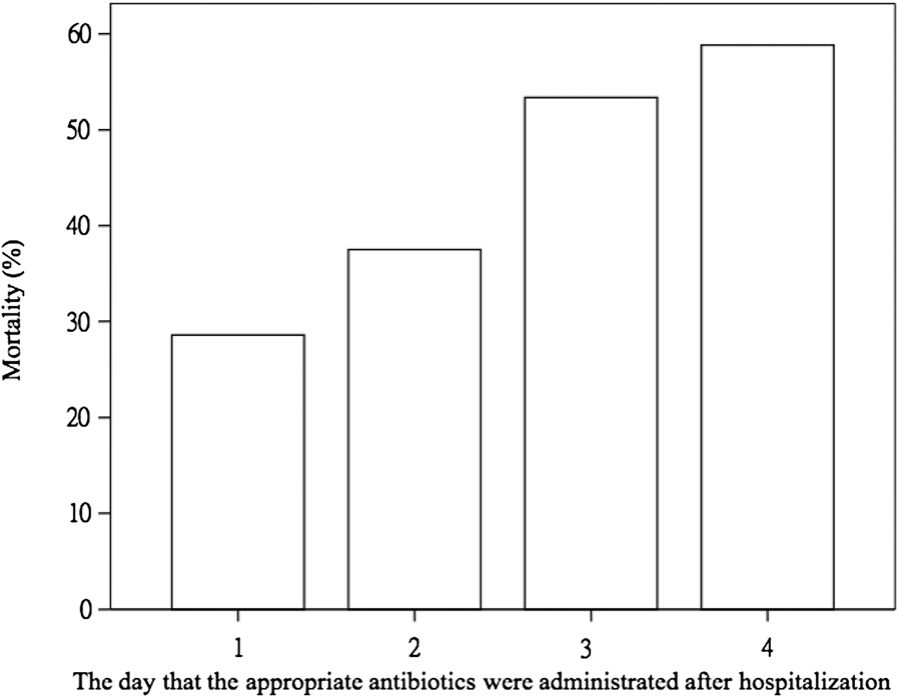
**Initial inappropriate antibiotic administration was significant associated with a higher mortality rate (Log Rank Test, *P*** **= 0.018).**

**Figure 2:**
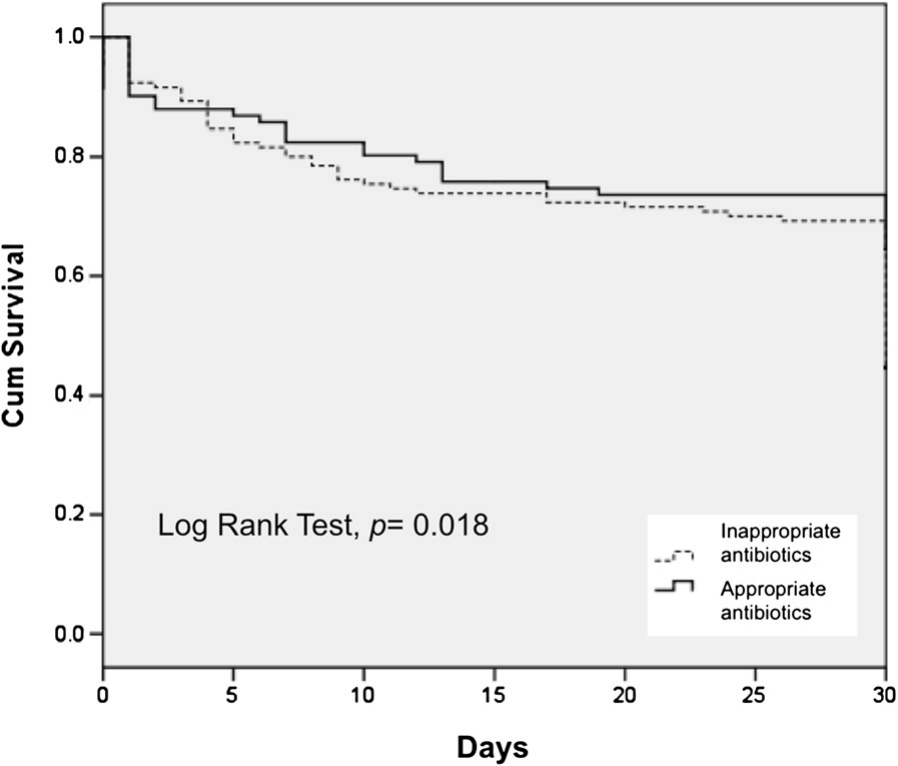
**In the inappropriate antibiotic treatment group, delayed adequate antibiotics administration led to higher in-hospital mortality (Chi-square test for trend, *P*** **= 0.07).**

After excluding CURB-65 parameters (confusion, BUN >20, RR > 30, SBP <90, DBP <60) and septic status (sepsis, severe sepsis, septic shock, MODS), the multivariate analysis revealed that the patients from nursing home (OR 0.267, 95% CI 0.091-0.970, P = 0.044), and normal WBC (OR 0.198, CI 0.068-0.574, P = 0.003) were significant for better outcome. In contrast, the patients with Initial inappropriate antibiotics use (OR 3.715, 95% CI 1.736-7.948, P = 0.001). In other hand, patients admitted to ICU (OR 4.241, 95% CI 1.620-11.104, P = 0.003) and needed ventilator use (OR 3.290, CI 1.034-10.466, P = 0.044) had the worst prognosis (Table 3). Furthermore, initial inappropriate antibiotic administration was significant associated with a higher mortality rate (Log Rank Test, *P* = 0.018) (Figure 1) and delayed appropriate antibiotic administration was associated with a trend of a higher mortality rate (*P* = 0.07) (Figure 2).

**Table 3:** Significant factors associated with mortality in multivariate analysis

	**OR**	**95% CI**	***P *** **value**
Nursing home	0.267	0.091-0.970	0.044
WBC 4000–10000 /uL	0.198	0.068-0.574	0.003
Initial inappropriate antibiotic use	3.715	1.736-7.948	0.001
ICU stay	4.241	1.620-11.104	0.003
Ventilator use	3.290	1.034-10.466	0.044

The pathogen related mortality rate and ratio of inappropriate antibiotic use are shown in Table 4. The most common pathogens in the study were *E. coli* (42 cases), *K. pneumoniae* (34 cases), MRSA (31 cases), and *Burkholderia cepacia* (23 cases). There was an outbreak of *B. cepacia* infection in the hospital between 2006 and 2007 [19]. The pathogens with mortality rates of more than 50% included MRSA*,* coagulase negative *Staphylococcus*, *Streptococcus pneumoniae,* Viridans *Streptococcus, Enterococcus faecalis, Klebsiella pneumoniae, Enterobacter aerogenes, Serratia marcescens, Citrobacter* spp.*, Hemophilus influenzae, Pseudomonas aeruginosa* and *Acinetobacter baumannii.* For the species with at least 10 isolates in the study, cases infected with MRSA and *Enterococcus faecalis* had more than 80% chance to receive initial inappropriate antimicrobial therapy.

**Table 4:** The pathogen related mortality and the ratio of inadequate antibiotics

	**Mortality**	**Initial inappropriate antibiotic use**	**Association of mortality and initial inappropriate antibiotic use**
	**N (%)**	**N (%)**	**(*P *** **value)**
***Gram(+) bacteria***			
*Staphylococcus aureus*	21/38 (55.3%)	25/38 (65.8%)	0.011
MSSA	1/7 (14.3%)	0/7 (0.0%)	-
MRSA	20/31 (64.5%)	25/31 (80.6%)	0.151
*Coagulase-negative Staphylococcus*	3/5 (60.0%)	3/5 (60.0%)	1.000
*Beta-hemolytic Streptococcus (Non Group A or B)*	0/1 (0.0%)	0/1 (0.0%)	-
*Streptococcus pneumoniae*	2/2 (100.0%)	0/2 (0.0%)	-
*Viridans Streptococcus*	2/3 (66.7%)	0/3 (0.0%)	-
*Enterococcus faecalis*	5/10 (50.0%)	9/10 (90.0%)	1.000
*Corynebacterium spp.*	0/7 (0.0%)	3/7 (42.9%)	-
***Enterobacteriaceae***			
*Escherichia coli*	16/42 (38.1%)	21/42 (50.0%)	0.340
*Klebsiella pneumoniae*	21/34 (61.8%)	23/34 (67.6%)	1.000
*Proteus mirabilis*	4/10 (40.0%)	6/10 (60.0%)	1.000
*Enterobacter cloacae*	0/1 (0.0%)	1/1 (100.0%)	-
*Enterobacter aerogenes*	3/6 (50.0%)	4/6 (66.7%)	0.400
*Serratia marcescens*	2/4 (50.0%)	3/4 (75.0%)	1.000
*Non-typhoidal Salmonella*	1/4 (25.0%)	2/4 (50.0%)	1.000
*Providencia rettgeri*	1/3 (33.3%)	2/3 (66.7%)	0.333
*Morganella morganii*	0/3 (0.0%)	1/3 (33.3%)	-
*Citrobacter spp.*	1/1 (100.0%)	1/1 (100.0%)	-
*Haemophilus influenzae*	1/1 (100.0%)	1/1 (100.0%)	-
***Glucose Non-fermenter***			
*Pseudomonas aeruginosa*	4/7 (57.1%)	5/7 (71.4%)	1.000
*Burkholderia cepacia*	8/23 (34.8%)	12/23 (52.2%)	0.193
*Acinetobacter baumannii*	6/8 (75.0%)	5/8 (62.5%)	1.000
*Stenotrophomonas maltophilia*	1/1 (100.0%)	0/1 (0.0%)	-
***Anaerobes***			
*Peptostreptococcus spp.*	0/2 (0.0%)	1/2 (50.0%)	-
*Propionibacterium spp.*	0/4 (0.0%)	2/4 (50.0%)	-
***Candida spp***	2/2 (100.0%)	1/2 (50.0%)	-

*MRSA* Methicillin resistant *Staphylococcus aureus*, *MSSA* Methicillin susceptible *Staphylococcus aureus.*

The most common inappropriate antibiotics used were cefazolin (57 cases, 43.5%), gentamicin (49 cases, 37.4%), and amoxicillin/clavulanate (35 cases 26.7%) (Table 5). For the other antibiotics, the rates were less than 10%.

**Table 5:** The frequency of inappropriate antibiotic use, categorized by the initial antimicrobial agents

	**Inappropriate antibiotics, N (%)**
Cefazolin	57 (43.5%)
Gentamicin	49 (37.4%)
Amoxicillin/clavulanate	35 (26.7%)
Ceftazidime	10 (7.6%)
Amikacin	8 (6.1%)
Ciprofloxacin	7 (5.3%)
Levofloxacin	6 (4.6%)
Vancomycin	5 (3.8%)
Piperacillin/tazobactam	5 (3.8%)
Piperacillin	3 (2.3%)
Meropenem	3 (2.3%)
Ertapenem	3 (2.3%)

Percentages are the likelihood of inappropriate antibiotic therapy for each specific antimicrobial agent.

The 30-day mortality rate and the analysis of factors associated with 30-day mortality were provided in the supplemental data (Table 6).

**Table 6:** The 30-day mortality rate in community acquired, nursing home acquired and nosocomial infection were 34.3%, 26.3%, 58.9%

	**OR**	***P *** **value**
Vascular diseases	0.419	0.011
Pulmonary infection	2.341	0.017
UTI	0.349	0.015
ICU stay	3.185	0.001
tracheostomy	0.362	0.003
Septic shock	2.810	0.004
MODS	13.714	0.012
Creatinine	2.6008 ± 1.6870 vs. 1.9296 ± 1.4225	0.014

The significant factors associated with 30-day mortality in univariate analysis were present in the following table.

*UTI* Urinary tract infection, *ICU* Intensive care unit, *MODS* Multiple organ dysfunction syndrome.

## Discussion

This is the first report about inappropriate antibiotic use and outcomes of cases of bacteremia in a community hospital in Taiwan. Multiple drug resistant microorganisms were frequently encountered in the island nation [20,21]. A high rate (59%) of initial inappropriate antibiotic use was observed, which is higher than most published studies [1,3-11] and have led to the poor outcomes. We observed that nursing home-acquired BSI cases had the best prognosis when compared to the hospital-acquired and community-acquired BSI groups. Patients with ventilator support and patients requiring ICU care were the independent predictors for inhospital mortality. Patients with normal WBC counts had better survival outcome. Delayed appropriate antibiotic administration was found to be associated with a higher mortality rate.

One of the major causes of inappropriate initial antimicrobial therapy was the ignorance of the possibility of antimicrobial-resistant organisms causing the infections. Only 16.2% (36/222) of the BSI in the study was community-acquired. Not surprisingly, cefazolin, gentamicin and amoxicillin/clavulanate were the most commonly used initially and inappropriate antibiotics when most bacteremia episodes were healthcare associated. These drugs are frequently prescribed empirically in Taiwan due to the low cost. The regulations of the National Health Insurance (NHI) authority in Taiwan may have contributed to this prescription habit. Almost all hospitals in Taiwan are included in the reimbursement system of the NHI. The amount of reimbursement for each hospital is usually fixed every year, and the NHI refuses to reimburse the medical expenditure when the expense is higher than the fixed amount [22]. All hospitals are requested to reduce cost; therefore, newer antimicrobial agents those may cost more are restricted from being used as the initial antibiotics in some hospitals. Even though most antimicrobial resistance surveillance studies show that the major pathogens in Taiwan are resistant to the first or secondary generations of beta-lactams and aminoglycosides [20,23,24], cefazolin, gentamicin and amoxicillin/clavulanate are still common choices of empiric antibiotics in community hospitals. Our result alerts clinicians to notice impact on patient outcomes due to inappropriate antibiotic administration. It is also necessary to improve the physicians’ appropriate use of costlier antimicrobials when patients with sepsis are at high risk of acquiring drug resistant pathogens. Furthermore, the time taken to change to appropriate antibiotic therapy influenced the outcome of patients with BSI in this study, consistent with other reports [25-27]. It is therefore necessary to evaluate BSI cases from time to time and adjust the antibiotics accordingly. This is especially important for patients in a critical condition with septic shock status and confused consciousness, both of which were prognostic factors for poor outcomes.

We observed that the patients with BSI from the affiliated nursing home had better outcomes in multivariate analysis. Possible reasons for this are that the patients from the hospital affiliated nursing home had adequate medical information. These referral medical information includes hospitalization history, previous culture report, and recent infection sites, these could help the clinicians to estimate the risk of BSI cases to have multiple drug resistant pathogens, thereby allowing for a rapid intervention in the early phase of the BSI at the hospital, and help the clinicians to choose initial appropriate antibiotics.

In this study, old age and having ventilator support were the most important underlying conditions influencing survival (Table 1). These findings are similar to those previously reported by Lee et al., in which they showed that the impact of inappropriate empirical antibiotic therapy on the outcomes of elderly patients with bacteremia was greater than that for younger adults [3]. A meta-analysis of BSI also revealed that inappropriate antimicrobial therapy significantly increased the odds of mortality in patients with ventilator support [9].

In this study, *E. coli, S. aureus, K. pneumoniae* were the three most common pathogens, which is similar to previous reports [6,28] with the exception of *B. cepacia* bacteremia. However, there was an outbreak of *B. cepacia* due to contaminated multiple-dose heparin vials [19]. In addition, our study showed that MRSA led to the highest inappropriate antibiotic use (80.6%) and the highest in-hospital mortality rate (64.5%) among all pathogens. This suggests that local community hospital physicians may not recognize the risk factors and consequences of MRSA infections, which can be corrected by identifying the local risk factors for MRSA and continuing physician education.

## Conclusions

In conclusion, a high mortality rate of patients with BSI was associated with a high rate of inappropriate antibiotic therapy in a community hospital in Taiwan. We observed a better prognosis in patients with BSI from a hospital-affiliated nursing home, whom were brought to the hospital with medical information. Efforts should be made to avoid the administration of inappropriate antibiotic therapy or delaying appropriate antibiotics administration in order to improve the outcomes of patients with BSI.

## Competing interests

All authors declare that they have no competing interests.

## Authors’ contributions

CYJ, TCC and PLL conceived and designed the experiments; CJY and YCC contributed to the acquisition of data; TCC and HLC contributed to the analysis and interpretation of data; CJY and TCC drafted the manuscript; YMT, MSH, YHC and PLL revised it critically and approved the final version. All authors had full access to all of the data (including statistical reports and tables) used in the study and take responsibility for the integrity of the data and the accuracy of its analysis. All authors read and approved the final manuscript.

## Pre-publication history

The pre-publication history for this paper can be accessed here:

http://www.biomedcentral.com/1471-2334/13/500/prepub
